# Health Care Experiences of Stateless People in Canada

**DOI:** 10.1177/23315024231200200

**Published:** 2023-10-13

**Authors:** Jocelyn Kane, Gezy Schuurmans, Miho Kitamura

**Affiliations:** University of Ottawa; Tilburg University; Desloges Carvajal Law Group

**Keywords:** statelessness, Canada, health, well-being, liminality

## Abstract

Statelessness in Canada is an emerging site of inquiry with recent investigations into its causes and consequences, focusing on legislative and policy analyses and the lived experiences of stateless persons. Yet, health care experiences generally and access to mental and physical health care in particular remain under-researched. This study attempts to bridge this gap by examining how statelessness impacts physical health, mental health, access to health care services, and overall well-being. To answer these questions, we conducted semi-structured interviews with stateless or formerly stateless persons to understand their views and experiences. The study reports on negative health outcomes in four broad areas:

- The limited ability of stateless persons (SPs) to access health care.
- Mental health challenges.
- The failure to treat health issues until they have reached a dangerous point and the reliance on self-care strategies.
- The negative impact of lack of status on four social determinants of health: employment, education, housing, and food security.

From these findings, the paper makes three arguments:

- Legal Status is a key determinant of health and lack of status leads to negative health outcomes.
- SPs heavily depend upon others for their life-needs, which can lead to exploitation and encourage forms of adaptive and negotiated agency.
- SPs in Canada experience a physical and mental liminality [a condition of uncertainty].

The paper concludes that Canada should recognize stateless individuals either as stateless or as Canadian nationals, and should implement a context-tailored institutional response to statelessness.

## Introduction

The right to the highest attainable standard of physical and mental health is internationally recognized and inseparable from the means to achieve the realization of other human rights (World Health Organization [WHO] 2017). Article 12 of the *International Covenant on Economic, Social and Cultural Rights* (UNGA, 1966) recognizes “the right of everyone to the enjoyment of the highest attainable standard of physical and mental health.” The ICESCR obligates state parties to promote this right for *everyone* regardless of “political, social or other status” (UN Committee on Economic, Social and Cultural Rights 2000, para 18). This right entails equal access to opportunities for good health, including the enjoyment of a variety of facilities, goods, and services necessary for the highest attainable standard of health (ibid, para 9), which must be available, accessible, acceptable, and of good quality (ibid, para 12). As part of the accessibility requirement, health facilities, goods, and services must be physically accessible and economically affordable to all, without discrimination, including to the most vulnerable or marginalized sections of the population (ibid, para 12(b)). The right to health also embraces socio-economic factors that promote a healthy life, and obliges states to create the conditions that allow people to lead a healthy life (ibid, para 4). This right extends to “the underlying determinants of health, such as food and nutrition, housing, access to safe and potable water, adequate sanitation, safe and healthy working conditions, and a healthy environment” (ibid, para 4).^1^

Despite these obligations, health care access is often restricted by the legal status of non-citizens,^2^ and is thus out of reach for millions of non-citizens around the world. Legal status is a marker of overall health and well-being, and a key determinant of health (Castañeda et al. 2015; Fleischman et al. 2015; Virgin and Warren 2021).

Canada is one of the state parties to the ICESCR that grants health care access according to legal status. A strong body of literature reveals that non-citizens in Canada experience negative health outcomes. Studies show that non-citizen residents have unmet health needs (Sou et al. 2017) including difficulties accessing health care (Hennebry et al. 2016), and while generally good upon arrival, newcomers’ health declines within a decade (Ng et al. 2005). Known as the “healthy immigrant effect,” this dec-line is experienced along ethnic and gender lines (Vissandjee et al. 2004), and socio-economic lines (Kim et al. 2013), especially for those who are temporary migrant workers (Preibisch and Hennebry 2011), refugees (Wanigaratne et al. 2016), and live-in caregivers (Carlos and Wilson 2018). Factors contributing to increased risk of poor health include difficulties in acculturation and adaptation (Kirmayer et al. 2011), and in accessing the conditions (social determinants) of positive health outcomes, including employment, housing, and education (Gushulak et al., 2011). Undocumented migrants experience barriers in accessing health services, and negative impacts on mental health resulting from fear of removal, social exclusion, and the inability to participate in society (Saad 2013; Magalhaes et al. 2010).

This article explores the health of the stateless in Canada. Statelessness in Canada is an emerging area of inquiry, mostly in the areas of law and policy. The health care experiences of stateless persons (SPs), and their access to mental and physical health care remain under-researched. Taking an approach that sees health as both an inherent right and as conditioned by determinants, the paper explores how statelessness impacts the physical health, mental health, access to health care, and the overall well-being of stateless persons.

The paper proceeds as follows. Section II presents an overview of statelessness internationally. Section III discusses statelessness and health in the global context. Section IV examines statelessness and health in Canada. Section V presents our methodology and Section VI our findings. In Section VII, we make three arguments concerning the nexus between health and statelessness. Section VIII offers a set of recommendations concerning statelessness and health rights for stateless persons in Canada.

## Statelessness

A stateless person is defined in the 1954 *Convention relating to the Status of Stateless Persons* (1954 Convention) as one who “is not considered as a national by any State under the operation of its law” (Art. 1, UNGA 1954). Estimates of the stateless population globally range from 10 million (UNHCR 2023b) to 15 million (Institute on Statelessness and Inclusion [ISI] 2014). Statelessness may occur when a person is unable to prove or establish nationality (de Chickera and van Waas 2017), within the context of migration, or in situ, when persons reside in their own country and/or have strong ties to the country in which they live (Vlieks 2017). Stateless persons may be refugees, legal residents, or undocumented. All, however, lack nationality. Thus, it is important to identify SPs and to establish policies and procedures that allow the (re)acquisition of an appropriate nationality (de Chickera and van Waas 2017).

Statelessness is often connected to discrimination against minority populations (Kingston 2017; see UNHCR 2017). In many countries women are discriminated against in nationality laws when it comes to their ability to acquire, change, or retain their nationality, or to pass on nationality to their children (Govil and Edwards 2014). Nationality may also be denied to particular ethnic, religious, or racial groups. Discrimination also limits access to citizenship as individuals may be entitled to the nationality of a state, yet unable to access it due to discrimination by state officials in administrative procedures (Jain 2022).

Stateless persons face several challenges related to the recognition of legal status and the ability to prove their identity. Because legal status is often necessary for accessing legal, social, and political rights, SPs can be denied access to health care, education, and the right to work. Furthermore, stateless persons often lack the ability to access routine life needs such as opening a bank account or marrying legally (UNHCR 2023a) and are at risk of exploitation (Foster et al. 2016).

Moreover, SPs face particular challenges in states that criminalize insecure legal status. For example, SPs may be arrested and detained in order to establish their identity, especially those who lack identity documentation, or when authorities believe an SP will not comply with the conditions of lawful residence (Bianchini 2020; Khan 2022). They also face unique challenges in proving that they are not a national of any state and may not be returnable to any state (Perks and Clifford 2009), and often face arrest and lengthy and arbitrary immigration detention awaiting effectuation of removal proceedings (Seet 2015; Chandran 2021), and in some countries even indefinite detention (Foster et al. 2016). Recognized legal status is therefore a key component in the facilitation of basic rights and protections of stateless people.

Guidance concerning the protection and prevention of stateless persons is set forth in internatio-nal human rights treaties, particularly the 1954 Convention and the 1961 *Convention on the Reduction of Statelessness* (1961 Convention), which are supplemented by UNHCR guidelines, such as the Handbook on Protection of Stateless Persons. The 1954 Convention offers a minimum standard for states that ensures stateless persons’ access to rights and articulates a stateless person status as a distinct legal status that states can implement. A legally recognized stateless person should be able to participate in society on equal terms and work toward acquiring a nationality. One mechanism designed to facilitate such recognition is a statelessness determination procedure (SDP), a tool that assesses whether a person is stateless and facilitates their regularization. Many countries delegate statelessness determination to immigration authorities (Gyulai 2014). Large stateless populations often have strong and long-established ties to the state in which they reside and have a reasonable claim to the nationality of that state (European Network on Statelessness [ENS] 2013). States can resolve these cases, in particular, by granting nationality (ENS 2013; UNHCR 2014).

Resolving statelessness is thus context dependent. Despite international guidance and evidence of good practices^3^ concerning how to identify, protect, reduce, and prevent statelessness, millions of stateless persons remain vulnerable to substantial human rights restrictions, including the right to health.

## Statelessness and Health

An emerging global literature on the health of SPs reveals a diverse set of physical and mental harms. The lack of identity documentation and proof of identity (and legal status) is particularly harmful as these are generally required for registration in national health service mechanisms (ISI 2023; Sköld 2023). Stateless people across the world suffer from unmet health needs resulting from the inability to access health care (Fokala and Chenwi 2014), dangerous working conditions, and malnourishment (McBride and Kingston 2014). Stateless persons have difficulty accessing preventative health care including vaccinations and birth registration, which contributes to child mortality (Kingston et al. 2010). Moreover, poor mental health outcomes are associated with stateless populations (Herberholz 2022). Specific mental health concerns include PTSD, depression, distress (Riley et al. 2017), anxiety, and risk of suicide (ISI 2023).

Such harms speak to the interconnections between legal status and various determinants of health; that is, the non-medical factors that can influence health outcomes (WHO 2023). Kingston et al. (2010, 3) observe that the right to health can be neither “understood in a vacuum [nor] realized in the absence of substantive and effective protections for a host of other human rights.” For example, poverty, lack of education, and insecurity resulting from precarious legal status negatively impact the health of stateless people (ISI 2023). Furthermore, statelessness negatively impacts a number of social determinants of health, including access to quality housing, sanitation, employment, and food security (Sköld 2023, 10–11). Koning et al. (2021) find that precarious legal status is a dynamic determinant that affects health differently at various stages of nationality recognition. The COVID-19 pandemic also exposes links between legal status and health outcomes. In particular, the effects of COVID-19 are compounded by the systemic barriers presented by statelessness, including poverty, malnutrition, and the lack of identity documents (van Hout et al. 2021), as well as increased gender-based violence (Chakraborty and Bhabha 2021).

Research on health outcomes as a function of structural and social conditions is complemented by scholarship on the impacts of what it *means* to be stateless. Conceptualizations of statelessness center on the impact non-nationality has on the ability to live a meaningful life. The exclusion generated by statelessness is seen to embody invisibility (Acciaioli et al. 2017) and isolation (Kerwin et al. 2020). Such a condition is considered an ontological harm in which the stateless person is deprived of the right to be seen and heard in society — fundamental human needs — and thus becomes an object to be cared for rather than a recognized subject (Parekh 2014). Lack of control over one’s life and freedom, and lack of access to resources needed to live threaten dignity and personhood (Kingston 2019).

Statelessness is also characterized as liminality, a supposed temporary condition “wherein a person becomes separated from [their] former identity and, through a rite of passage, takes on another identity” (Belton 2015, 911). Statelessness as liminality tends to be grounded temporally as the stateless person navigates their past, present, and future interactions with status, and spatially as they are bound by socio-legal exclusions from mobility (Parsons and Lawreniuk 2018). It is a condition of uncertainty as one navigates “rupture” from their homeland amidst possibilities for future resettlement (Gupta 2019). A tension emerges, however, when liminality is considered a product of state action. Belton (2015) observes that stateless people are sometimes forced into a liminal space through legal and socio-political discrimination in their homeland. Such in situ displacement contributes to a psychosocial liminality where stateless persons live in a condition of rejection and confusion as to where they belong (ibid.).

Yet, SPs also resist the conditions they face. For example, individuals navigate barriers to health care by taking out loans from community members to pay for health services, visiting unlicensed or untrained practitioners, and by choosing to give birth outside of hospitals and medical supervision (Zaman et al. 2022). By this view, membership does not operate on a linear statelessness → citizenship trajectory, and steps taken to navigate statelessness are fluid, negotiated, and contingent upon social and structural conditions (Redclift 2013; Sigona 2016; Brinham 2019).

## Canada, Statelessness, and Health

There is an emerging body of literature on the causes and consequences of statelessness in Canada, and gaps in Canadian legislation and policy. Although Canada has ratified the 1961 Convention, it is not a signatory of the earlier 1954 Convention. It has taken the position that the Canadian Charter, *Immigration and Refugee Protection Act* (IRPA) and the *Citizenship Act* already provide protections for stateless persons in Canada (Brouwer 2012). Substantial protection gaps exist, however, for non-refugee stateless persons in the areas of social housing, public education, health care, social assistance, social security, identity papers, travel documents, expulsion, and naturalization (Erauw 2015). Furthermore, Canada’s *Citizenship Act* and IRPA do not define a stateless person. The IRPA defines a foreign national as “a person who is not a Canadian citizen or a permanent resident and includes a stateless person” (s. 2 Government of Canada n.d.-b). Capturing stateless persons in this catch-all category of “foreign nationals” evades the identification and recognition of statelessness by presuming nationality of a foreign country when stateless persons are nationals of no state.^4^ Finally, Canada does not have a specific process for statelessness determination, and it is unknown how many stateless persons reside in Canada (Kane 2019).^5^

While Canada has a universal health care system available to any citizen or permanent resident who fulfills specific residency requirements (Government of Canada 2021), legal status and residency determine health care access, which varies across jurisdiction. In the province of Ontario, for example, those holding a work permit must be working for an Ontario employer full-time for at least six months (Government of Ontario 2023), though it is not clear whether Ontario provides health insurance coverage for stateless persons issued a work permit (Erauw 2015, 81).^6^ Ontario does not provide coverage for international students studying in Ontario, who must pay for coverage through the University Health Insurance Plan (2023). For protected persons — including those who claim and are granted refugee status, temporary resident permit holders who are victims of human trafficking or domestic violence, and immigrants in detention — the federal government grants health care through its Interim Federal Health Program (IFHP) (Government of Canada 2023a). IFHP coverage is limited and temporary, and the content and duration differ across these groups.^7^ No health care infrastructure exists for stateless individuals outside of these categories of protection (Erauw 2015, 71–2), leaving undocumented stateless persons without care (Chabot 2021, 146).

A growing but fragmented scholarship looks at some health care experiences of stateless persons in Canada and shows that access to physical and mental health care for stateless persons is restricted. Stateless persons disproportionately experience gaps in access to reproductive health care and abortion in Canada including financial, linguistic, and proximity barriers (Chabot 2021). Stateless detainees are denied access to mental health services, placed in solitary confinement (Gros and van Groll 2015; Budlakoti 2021), and subject to poor recognition and support of psychosocial disabilities, an overall experience described as “traumatizing,” “torture,” and “hostile” (Human Rights Watch and Amnesty International 2021). Stateless people in Canada also experience mental health challenges including hopelessness, addiction, and suicidality (Kane 2019). Furthermore, difficulties in securing work permits can lead to informal employment and labor exploitation. Such circumstances can negatively impact standards of living and access to basic needs including housing and nutrition (Kane 2019). It is against this backdrop that we embark on a focused exploration of the physical and mental health experiences of stateless persons living in Canada.

## Methodology

This pilot study is an analysis of data collected on the nexus between health and well-being, status, and belonging. The study carried out qualitative semi-structured interviews with individuals residing in Canada who were stateless or formerly stateless to explore their views and lived experiences. This study is exploratory rather than representative as it aims to paint a modest and preliminary picture of the health of SPs in Canada, and is a starting point from which more comprehensive and generalized studies can depart. Our sample size was small and intended to evaluate our pilot research methods and identify community and organization stakeholders for future scaled-up research opportunities. Ethics approval for this study was received by the University of Ottawa Research Ethics Board, file number 09-17-05.

To approach participants, we relied on two sourcing methods: circulating project information emails through the listservs of service provider organizations and our professional networks, and snowball sampling thereafter. All participants provided written informed consent. Between November 2017 and May 2018, we conducted six semi-structured interviews involving stateless or formerly stateless persons. All participants lived in Canada and spoke English. Three were female and three were male. Half were parents and half were in their early adulthood. Participants were either not recognized as nationals of any state, could not prove the details of their birth, or had trouble accessing national identification documents due to state secession. Four had no legal status in Canada, one was a recognized refugee, and one was an asylum seeker awaiting status determination decision. The recognized refugee was a permanent resident and thus had access to full provincial health care. The asylum seeker possessed IFHP coverage.

Participants were asked a series of questions that addressed two broad topics: how stateless residents characterize their belonging to Canada, and the impact of belonging to Canada on their self-assessed physical and mental health and health care access. Participants were encouraged to articulate belonging in their own terms. We asked questions about day-to-day experiences of nutrition, employment, and housing; relationships with others; accessing health care including mental, emergency, and specialist services; and feelings, both generally and for the future. We focus in this paper on the health experiences of participants.

To reduce the potential of interviewer bias, three members of our research team carried out interviews, and a different set of three took part in the analysis and writing processes. Only one research team member took part in all interviews, analysis and writing phases. The interviews were tape-recorded and transcribed, and transcripts were sent to participants for approval of content and privacy concerns. Participants were given the opportunity to redact content from the transcripts. Once approved, transcripts were input into NVivo software for organization, open and axial coding, and analysis. All names are pseudonyms.

## Findings

Our findings are grouped into four overlapping themes that address access to health care services, mental health, self-care and coping strategies, and social determinants of health. The legal predicaments of our participants varied across and within migratory and in situ contexts. For example, Hank and David were raised in Canada since infancy and could not prove nationality for decades; only later in life were they granted Canadian citizenship. Miryam was brought to Canada as a child on a valid visa yet found herself unable to access the nationality of her home country after its state secession. Neeha found herself in a similar situation. Sergio is a recognized refugee yet cannot prove the validity of his birth registration and remains a stateless person with no travel documents, despite holding permanent residence in Canada. Valentina is not recognized by any state as a national, but if recognized as a refugee she will become a stateless permanent resident.

While we detail various factors related to the health experiences of our stateless participants (SPs) below, at the outset we note some of their physical ailments to help contextualize the diverse experiences in accessing health care, coping with various stressors, and overall well-being. Participants faced some major illnesses, chronic pain, and injuries, almost all of which went untreated for a period of time or were not treated at all. Neeha had chronic pain for ten years due to ulcers in her bladder. Miryam could not go to a gynecologist despite experiencing pain due to ovarian cysts. Both had initial imaging carried out but were unable to follow up with treatment. Valentina had anxiety induced breathing troubles, panic attacks, and faints, as well as digestive challenges. Hank had several heart attacks and had not received adequate care to recover and was left partially disabled. Sergio also had a heart attack, suffered from back problems, arthritis, high blood pressure, and is pre-diabetic. David suffered a shoulder injury which was left untreated, and both him and Hank had to stitch themselves up after wounds. The table below outlines the demographic, legal, and health profiles of our participants.

**Table:** 

Name	Gender	Legal Status in Canada	Cause of Statelessness	Health Care Access	Physical Ailments
David	Male	No legal status	Could not prove place of birth	No; uninsured	Shoulder injury, open wounds
Hank	Male	No legal status	Could not prove place of birth	No; uninsured	Heart attacks, open wounds, tooth abscess
Miryam	Female	No legal status	Could not access national identification documents due to state secession	No; uninsured	Ovarian cysts
Neeha	Female	No legal status	Could not access national identification documents due to state secession	No; uninsured	Bladder ulcers
Sergio	Male	Permanent resident; recognized refugee	Could not prove validity of birth registration	Yes, OHIP	High blood pressure, back pain, arthritis, pre-diabetic
Valentina	Female	Asylum seeker awaiting status determination	Not recognized as national of any state	Yes, IFHP	Digestive, breathing troubles, fainting

### Theme 1: Access to Health Care

Most participants were limited in their ability to access health care as they were ineligible for government health insurance plans. Therefore, they had to pay out of pocket for these services, which caused them significant financial burden and often prevented treatment. David spoke of the time he needed to get stitches but was unable to pay for treatment and his wound was left to heal on its own. In reference to another incident, he explained:

It’s not even worth it to go to the hospital for something that a normal person wouldn’t think twice about going to the hospital for. You can’t do that if you’re stateless because it costs too much. It’s not feasible. When I thought I dislocated my shoulder [. . .], I went to the hospital . . . and they couldn’t do anything for me. The lady was so upset about it too. There was nothing she could do about it. She said “you’ve just got to pay this much” and it was a ridiculous amount of money. And I [said] “ah, no thank you” and I left. And she [said] “you can’t live like this.” And I [said] “well I don’t really have a choice.”

Reflecting on a previous hospital visit where he was billed, Hank noted he does not seek health care unless it is an emergency. Although Neeha has the name of a specialist who can assess her chronic pain, she spoke of her inability to proceed with treatment because of the cost. When asked about accessing mental health services, Miryam stated she once saw a psychiatrist, but all she could think about was the money.

Neeha and Miryam experienced a distinct barrier to accessing health care. Their access was limited by family members whose approval was needed before health care could be sought. Miryam recalled that she visited the emergency room, which billed her father $1,800: “Yeah, we have to wait a certain point to make sure we’re sick because it’s a lot of money.” Neeha also expressed her desire to see a health care professional and not have to wait until it was an emergency. She revealed how her husband controls how and when she can seek health care services:

If I could, I would really like to go to the doctor when I have the flu so I don’t have to wait for a month to prove to [my husband] that I’m really sick and I really need it. I would rather do it on time because this torture doesn’t make sense. But it’s his money and I can’t tell him what to do with his money. So, we wait until [I] can’t take it anymore and he takes [me].

Miryam and Neeha both took preventative measures to reduce the risk of having to go to the hospital that include not walking outside in the winter or going on ski trips because of the risk of falling and breaking a bone. Neeha expressed: “When you become a citizen is when you can do stuff like that. [Then] you can put yourself in danger but right now we can’t afford . . . to end up in the hospital again.”

### Theme 2: Mental Health

Participants described a diverse set of mental health challenges. These included depression, anxiety, stress, a range of negative emotions and feelings, a poor sense of self, and relationships with others characterized by dependency and distrust.

Staying at home being cooped up in that room, it’s got to the point where I want to scream. I’m aggravated. . . . I’m sad, just really depressed. (Miryam)

I only have feelings when I have the panic attacks. I feel the fear. I feel bad. But that’s it. But like, having positive feelings? I don’t know how to feel that anymore. (Valentina)

[My child] said “cmon mom, in all the pictures you are serious, smile a bit.” And I smiled and I started crying. We don’t laugh anymore. I don’t smile, I don’t laugh anymore. We just worry. It’s a constant worry. (Neeha)

Our SPs provided a startling picture of a range of negative emotions related to their lack of status. They reported feeling abandonment, anger, deception, disrespect, embarrassment, mental exhaustion, fear, frustration, guilt, hopelessness, humiliation, loneliness, powerlessness, sadness, shame, and shock.

#### Sense of Self

SPs expressed that they feel as if they do not exist, are less than human, are wasting their lives, are dead or invisible, and are useless and hopeless.

At some points I was doubting if I exist. If I exist in this world. Am I a person? Like all people? (Valentina)

And this makes me feel bad about myself. I’m useless. I’m helpless and useless. I am a waste of life. My life is wasted. (Neeha)

I lost . . . I lost hope . . . I don’t . . . I don’t care no more. Accept the consequences. (Sergio)

Participants expressed that their circumstances lead to pressures to become someone else to survive such as adopting criminal behavior or hiding the reality of their status from social networks. Hank explains succinctly: “It all boils down to choices to how you’re going to survive, what you’re going to do.” David explained the tension he faced between his morals and his survival instincts and stated:

I felt I might have to change who I was in order to survive. When you’re raised, everyone knows what right and wrong is. I had these ideals, I was my own person and it was getting to a point where I was going to have to ignore those, ignore my perception of what right and wrong is and basically turn into someone who I’m not, who I don’t want to be, in order to be able to survive. What are your options if you can’t get work? You got to sell drugs. (David)

#### Relationships With Others

Lack of status impacted the relationship that participants had with family, friends, and neighbors. Due to their exclusion from many aspects of society, SPs were dependent upon others. Neeha depended on her husband for money to access health care services. For several years, David relied on his friends for housing. Miryam relied on her father for access to health care, medicine, and finances. This sense of dependency also impacted their sense of self. As Neeha expressed:

Right now, my life, I don’t live my life. I live my husband’s life. [. . .] He [is not stateless and] has a normal life, he works. He has colleagues. He has friends. I can’t afford to have friends because of my emotional state.

That’s the situation [I’m] in now. Having to ask someone for clothing, for food, for money. (Miryam)

In other instances, SPs were stereotyped as liars when others found out about their statuses. Miryam, for example, decided to end her relationship with her boyfriend when his parents warned him that she may want to marry him for legal status.

How do you bring this up in a conversation to somebody? [. . .] Some understood, some I haven’t spoken to since it came out because they just called me a liar. (Hank)

David describes how he avoids conversations:

Talk comes across about what you’re doing and your plans for life and what not, like where you’re going. That was definitely a big issue for me, avoiding those questions, because being a normal person who can function in society, we talk about stuff, have a conversation. But when [. . .] they’re stateless, they’re not a member of that society.

All SPs experienced isolation from their peers. Whereas their friends and families were achieving major milestones in life, such as getting a driver’s license, a promotion at work, or buying a house, SPs were unable to achieve these things.

I remember one time, I think it was grade 10 or whatever, in a personal planning course we took, [. . .] we did a [personality test] . . . and I refused it. You know, what’s the point of doing this? I’m not going to be able to do any of this stuff. What’s the point? (David)

“I choose rather not to be or speak with anyone. But at the same time I feel so lonely [. . .] I’m really lonely. I would like to have colleagues and friends and a home, finally a home. (Neeha)

#### Fear and Trust

The relationship between fear of removal or detention and trust in authorities is complex and was not felt in the same ways by all participants, but most did have a fear of being “found out,” detained, or deported.

So, we’re just scared of getting in any situation. I’ve had so many opportunities to do so many fun things but I know I will never do them because I don’t want to get deported. (Miryam)

Anytime I happened to be in the wrong place at the wrong time and the police are asking for ID, that’s when the terror comes, that’s when [you say] OK, is this the time they’re going to go “We don’t know who you are so we’re locking you up?” (Hank)

The fear of discovery prevented some SPs from seeking police assistance.

I had an experience with a friend where I was in a bad situation and I couldn’t even go to someone and tell them because I was too scared that I could get into trouble for being hurt. (Miryam)

[One day the school] told me [my child] was attacked, the [school] principal asked us “do we want to call police?” and I said “absolutely not.” I absolutely would if I was a citizen. (Neeha)

Yet, for David, the fear of discovery by authorities was a kind of solution to his predicament. David stated he was confident that should he have an encounter with police he would either be treated well or let go without investigation or detention. David also saw in a police encounter the opportunity for recognition, “an acknowledgement that I existed,” and he engaged with immigration authorities with a similar attitude:

I phoned up Citizenship and Immigration Canada a few times and I [. . .] told them exactly my situation [. . .] I wasn’t scared they would deport me because I didn’t care. That’s where I was at in life. I didn’t see any future for me. I had to do everything I could. I put myself out there. I put myself in front of them. I said, basically, “I’m here. Do something.” It wasn’t a fear for me at all.

Trust in immigration authorities, however, was otherwise significantly lacking. All participants lacked confidence that immigration authorities knew how to effectively handle a “stateless case,” or that they could reasonably provide insight on steps that could be taken in filing status related applications, or in their understanding of statelessness in the first place.

### Theme 3: Self-Care and Coping Strategies

In the absence of care for both stateless persons’ mental and physical health, participants adopted coping or self-care strategies to help manage physical health care needs including seeking alternative or informal health care, providing their own health care, and avoiding getting sick. Coping with mental health challenges featured prominently in participants’ accounts and included intentional social isolation and managing expectations by assuming negative outcomes.

First, SPs sought alternative or informal health care, which included seeking care with a registered practitioner without being legally eligible for it, and sharing medicine with or using the prescriptions of family members or spouses. Some participants found mental health support in spirituality. Neeha and Sergio both expressed that spirituality can help relieve the financial burden of seeking clinical support.

Second, when faced with ineligibility for health care, many SPs tried to provide their own. Valentina used tape to bandage wounds. Hank took a bus instead of an ambulance to get to the emergency room, gave himself stitches after researching instructions in a medical journal, and pulled out his own tooth. He explained:

I never went looking for it because I knew it simply wasn’t available for someone like me. Mental health services, dental, anything to deal with your health, you deal with it yourself unless it’s an absolute emergency . . . I’ve gone because I’ve had an abscessed tooth, and no they can’t help you. So I go deal with it myself, get rid of an abscessed tooth myself, and that’s what I’ve had to do.

Third, SPs avoided traveling or participating in events to avoid possible injury, which led to a feeling of physical and social isolation. As Neeha expressed: “I am like a prisoner. I’m stuck in the house. And I don’t have friends for a reason.”

Participants developed strategies to cope with a variety of mental health challenges. One prominent coping strategy was social isolation. Participants often pre-emptively avoided relationships or even simple conversations with neighbors as they continuously had to explain their situations, including that they had not yet resolved their legal precarity or received negative reactions to explanations of their stateless status. Consequently, they avoided social interaction. All SPs felt isolated at some point. Like Neeha, Hank kept struggles surrounding his status hidden from his family and friends. Sergio explains his experience when he was asked whether his status was resolved:

“Oh! I’m working on it.” “Oh, not yet!” If I say one word, it becomes twenty from them. They say, “why not? What happened?” So, I don’t want a conversation. I don’t want that. So, it affected me even to really talk to my friend or talk to anybody or even my neighbor. I start hiding, seriously.

Another coping strategy was a form of expectation management. Participants dealt with frequent uncertainty and disappointment about their futures over which they had no control. Accepting that there was no hope or that things were not going to work out in their favor was a way to cope with the challenges presented to them. These expressions resemble hopelessness and depression but were also a way to cope with uncertainty. Accepting that there was not going to be a positive resolution provided a sense of stability, even if this meant acceptance of a negative reality or a potential negative outcome.

I’m getting to that point where it’s not that I want them to deport [me]. I just want to know. (Neeha)

It’s too late. I really have no faith in the system. [. . .] You know we’re talking about 30 years. What faith are you talking about? There’s no faith. I lost faith. (Sergio)

Another way of dealing with uncertainty and accepting reality is to “forget about things” and live day by day.

I’d rather just sit here and listen to music. Music you know just . . . it made me forget a lot of things. (Sergio)

I gave up. I give up. I’m just living my life [. . .] one day at a time, a leaf in the wind, and let’s see what happens. (Hank)

### Theme 4: Social Determinants of Health

Lack of status for stateless persons negatively impacts four social determinants of health: employment, education, housing, and food security.

#### Ability to Secure Employment

Most of our SPs referenced how their lack of status impacted their ability to secure employment as they were unable to acquire a work permit. Some of the participants spoke of their dependency on employers who were willing to hire them illegally.

You’re looking at the $50 that you [made] for cleaning the shit off a mattress in a hotel room for five hours. Those are some of the most disgusting jobs because nobody wants to do it but they’re going to pay me a hundred bucks to do it? OK, I’ll go do it. (Hank)

David commented:

You are relying on the good will of other people to hire you illegally. So, it’s definitely not stable at all. If they’re going to snap their fingers and screw you over there is nothing you can do about it.

Many participants expressed a strong desire to work, acknowledged their competency and capability to work, and viewed working as contributing to society. For example, Hank spoke of being qualified for certain jobs due to his experience, and lamented the unfairness at his ineligibility to work at these jobs. Neeha, felt like she couldn’t contribute anything to society as someone without status who could not work:

Working makes me feel good [. . .] it was never about money. It was about me being able to work so if I have to work as a cashier or in a Costco, I won’t care. I will feel good. I will finally feel good about myself [. . .] [I] want to work and contribute and stop dreaming about it.

Neeha also described how she could not volunteer because the application to do so required a police record check: “And so I stopped asking to volunteer after that.” Miryam expressed similar sentiments about her desire to work. She volunteered at an organization, and was offered a job but she couldn’t accept the position. She commented:

We’re capable, we’re intelligent people. We can work and we should be allowed to work. I think it’s just really unjust.

#### Ability to Access Education

Participants had varied experiences with accessing education. Those who attended school could do so because of the good will of administrative and teaching staff who permitted their enrolment without identification documents. After being rejected by many high schools, Miryam was eventually enrolled due to a principal who decided not to record or disclose her status. David was homeschooled and could go to high school and college only because both institutions allowed him entry despite not possessing identity documents.

There was also a strong desire to learn. Miryam dreamed of becoming a lawyer in Canada and expressed her powerlessness at not being able to do so. Hank noted that ever since his father gave him an Oxford dictionary, he would sneak into libraries whenever he could.

#### Housing and Food Security

Most participants spoke about difficulties in securing stable housing. Accessing housing was limited for reasons of affordability and the requirement to provide identification documents to potential landlords. These factors presented a risk of homelessness, making SPs dependent on the good will of others to meet their temporary housing needs.

David noted that to rent an apartment, landlords required certain documents such as records of past housing and a record of employment, which were difficult to obtain. He recalled being denied housing numerous times because he didn’t have the required documents, and sensed that landlords were uncomfortable with his lack of status. Hank spoke of stable housing as out of reach:

We may have a friend that we can sleep on their couch for a couple nights, but to go rent a place? No. Can’t dream about a house, you’ll never have a house . . . Not even a little shack that we can call our own. It’s impossible.

Hank also noted that his precarious employment sometimes prevented him from eating for days because he was unable to afford food. He described how stateless people may have to resort to stealing food:

You’re starving to death, you can’t get a job because it’s illegal for you to be alive — there’s a pack of hamburger buns there at the grocery store, I’m starving, I need to take them. I’m sorry but I have to, I have to eat — that’s different. That’s what stateless people have to deal with. “Where’s my next meal coming from?”

## Discussion

Study participants provided a first-hand account of what lack of eligibility for health care looks like on the ground. Their experiences prompt us to make three arguments. First, our research makes it clear that legal status is a key determinant of health in Canada. Though based on a limited sample size, this result is consistent for all participants regardless of their ability to access other forms of protection, or whether their legal status moves between in situ and migratory contexts. Spanning diverse indicators in four broad areas — access to health care services, mental health, self-care and coping strategies, and social determinants of health — statelessness is linked to negative health outcomes in line with global trends (ISI 2023; Sköld 2023).

Most SPs were limited in their ability to access government-subsidized health care causing significant financial burden that often prevented treatment. In the absence of services, the health issues of study participants were left to spiral to a dangerous point before they were addressed or were not treated at all. As a result, study participants resorted to self-care strategies. In addition, statelessness negatively impacts employment, education, housing, and food security, thus confirming the role of legal status in health outcomes. Here, Kingston et al.’s (2010) claim that the right to health cannot be separated from other substantive rights finds currency in the Canadian context.

This study broadens our understanding of statelessness and health in Canada by exploring the nexus between status and health as SPs age, consider life goals, and interact with immigration processes. Our participants’ experiences also illustrate that the legal statuses of stateless persons in Canada are not fixed but can change throughout life in both in situ and migratory contexts (Koning et al. 2021). Our findings thus add statelessness to the corpus on non-citizen vulnerability in Canada and confirm that lack of nationality has egregious effects on health and well-being, in ways that mostly align with other non-citizen groups.

Our second argument concerns the relationship between dependency and statelessness. For stateless persons, dependency is a double-edged sword. On one hand, dependency is widespread and incurs the risk of exploitation. On the other hand, it can encourage adaptive and negotiated agency.

From their spouses to friends, participants described depending on others for shelter, general life-needs, access to medicine and health care, employment, legal assistance, and keeping their legal status private. Hank took short-term low paid jobs; both Neeha and Miryam needed to prove to family members that their ailments were significant enough to warrant the cost associated with treatment; and both Miryam and David depended on the good will of school principals to enroll them in secondary school. At times, study participants faced the risk posed by revealing their status and relied on others to break the rules for them. Dependency also takes place institutionally, as evidenced by the need of SPs to address their legal predicaments with immigration authorities. Study participants depended on officials to guide them in taking the necessary steps for a resolution of their situation, while having little trust in authorities’ knowledge of statelessness generally or in the Canadian context.

Research on the nexus between statelessness and exploitation in Canada is wanting. Yet as this study reveals, the risk of exploitation is real. In this way, statelessness might be seen as another example of non-citizen vulnerability based on legal status. Statelessness has a direct impact on the ability to pursue health and well-being autonomously, and to meet immediate and future life-needs.

Yet our data also shows that stateless people in Canada have developed mental and physical coping strategies and employed efforts to resolve their legal cases, revealing a picture of empowerment despite dependency and associated risks of harm. The agency that emerges here is not black and white nor clearly “positive,” but rather strategic and at times disturbing. The idea of “taking control” of one’s life is key to those we interviewed in two ways. First, study participants described taking their health into their own hands when they were unable to access professional health services. Hank self-diagnosed his tooth infection, researched how to address it, assessed the risks involved, carried out the procedure, and dealt with the aftermath. Upon learning of the cost of emergency treatment for his injured shoulder, David weighed his options on the spot — to endure pain and possible complications, or face a hospital bill — while a concerned nurse awaited his decision.

Second, when faced with the risk of exposure, SPs avoid intimate relationships, offer false status updates, and do not seek needed assistance from police and other authorities. To avoid the stresses involved with being unrecognized by society, Sergio reported avoiding conversations with neighbors by moving from his porch to inside his house. Neeha chose not to make friends and stayed silent when passing strangers. Miryam isolated herself in her room. Such strategies are meant to prevent stress and reduce the potential of statelessness-related physical and mental challenges. Stateless people in Canada must constantly navigate risks and opportunities, demonstrating that vulnerability and empowerment are not fixed but rather fluid in response to changing physical and mental health needs. In this way, as SPs respond to restrictions on their health, the body itself becomes a place of both contestation and potential.

We observe that adaptive and negotiated agency takes place in a vacuum of other options, which leads us to our third argument, that stateless persons in Canada experience uncertainty in legal and social spaces, but also in the body and the mind, a physical and mental liminality. Despite having meaningful relationships, legal exclusion from society contributes to the risk of identity and behavioral change. SPs adopt survival practices that conflict with their values, resulting in a psychosocial liminality where they take on another identity (Belton 2015). To navigate a restricted world, they must find other ways to survive: David considers a life of crime while Hank works illegally and steals food to nourish himself. All the study participants reported thinking about or taking action to harden their personalities, withdrawing from some social spaces, and entering new and potentially dangerous spaces. Living as a stateless person in Canada is a balancing act between retaining a sense of self and purposely trying to lose it at the same time. This metamorphosis manifests where positive feelings are limited, fear is constant, anxiety is omnipresent, all of which contribute to feelings of uselessness and non-existence. Our participants seek formal recognition for its legal grounding but also for the acknowledgement that they *exist*. Here, statelessness is indeed “a question of non-existence or obstruction from existing” (Eliassi 2015, 24).

The harms we noted above pose significant risk to one’s physical health but also induce a particular type of liminality where SPs do not know if they will heal or endure further physical harm. Indeed, pulling out one’s own tooth is a sign of taking control but the potential of other harms follows: blood loss, infection, unanesthetized pain, and damaging other teeth, the jaw, the gums. One who takes these steps must accept the risks, monitor healing, and assess whether further care is necessary. In taking their health into their own hands, SPs are forced to engage in an inhumane experience. Here, non-recognition is compounded by the liminal space within which an ill or injured stateless person must navigate treatment and risk of exposure. In situ displacement (Belton 2015) moves beyond confusion about where one belongs and into the body where living a meaningful life is conditioned by one’s physical well-being.

We note some limitations to our study. A scaled study of this kind could yield more fulsome and disaggregated findings. For example, our research focused on the relationship between statelessness and health with the aim of highlighting statelessness as a sub-group with special needs within the larger group of non-citizens in Canada, and we did not specifically explore the intersections of health and gender, race, religion, Indigeneity, or ethnicity. Furthermore, scaled research of this kind would cast a wider net on conditions of statelessness not explored in this study including activism and advocacy, inter-provincial and -territorial variations in populations and service provision, and could provide a more robust data set upon which governmental collection of health data by legal status could be based. Moreover, comparing health care experiences to other non-citizen groups was outside the scope of this paper. Further comparative research could be conducted on health experiences of different non-citizen groups, and on the effects of overlapping statuses both within and across them.

## Recommendations

We have shown that despite Canada’s obligations under the ICESCR to respect, protect, and fulfill every person’s right to the enjoyment of the highest attainable standard of health, Canada’s health care system is predicated along the lines of legal status. Nonetheless, having signed the Los Angeles Declaration on Migration and Protection in June 2022, Canada agreed to strengthen frameworks for international protection for migrants, including stateless persons (Office of the Prime Minister of Canada 2022). This, alongside the recent move to explore the regularization of undocumented people in Canada (Government of Canada 2022), presents an opportunity to officially acknowledge that statelessness exists in this country, respond to calls for action, and comply with international obligations.^8^ In this light, we make two concrete recommendations that simultaneously address health and statelessness through which Canada can make a substantial difference in the lives of stateless persons.

First, in the absence of an overhaul to the Canadian health care system where everyone is granted health care irrespective of residency status, Canada’s immigration and nationality systems should be reformed to recognize, through a distinct protection stream, SPs as stateless or as Canadian nationals. Such recognition would then facilitate access to health care through provincial or territorial coverage, which would reduce the marginalization of SPs with no legal status who are denied equitable — including economically affordable — access to health care. As the experiences of our participants Valentina and Sergio, who have access to IFHP and OHIP (respectively), make clear, non-refugee stateless persons face disproportionate health related harms, one of which is the cost of health care for the uninsured. Legal recognition would remove barriers to accessing health care and render care economically accessible, absolving stateless persons of the need to rely on self-care strategies or “back alley” treatment. The ability to access health care would also remedy a host of other health care needs not reported here: pre- or post-natal care, broken bones, preventable and treatable illness such as diabetes induced wounds or cancer, and routine check-ups and vaccines.

Moreover, given Canada’s acknowledgement that determinants of health play a role in well-being (Government of Canada 2023b), and the claim that resolving statelessness can improve such determinants (ISI 2023), legal recognition is a poignant step in addressing the connections between health and status. The right to work legally, for example, is instrumental in stateless persons’ well-being as it is deeply connected to several other determinants of health. The right to work would enable stateless persons to legally pay for housing, food, and other goods and services, and reduce the need to steal for food or participate in informal economies. Legal recognition would also provide a more accurate picture of Canada’s workforce and labor industries and inform Canada’s recent acknowledgement that undocumented workers contribute economically *and* are vulnerable to employer abuse (Government of Canada 2022). Removing the need to consider illegal activity and depend on others would thus greatly support stateless persons as agents and reduce their risk of exploitation.

Recognition of stateless status or of nationality can also facilitate healthy conditions of life related to one’s sense of self and relationships with community. Recognition can solve uncertainties in life that our stateless participants detailed and the stress, anxiety, and panic accompanying that uncertainty. Recognition would give a sense of resolve to often years of ambiguity where both rights and course of action are in flux, and help stateless persons move from a situation of limbo to one of permanence where they can escape fears of detention. Recognition can also promote well-being by enabling stateless persons to publicly nurture the strong ties to Canada and desire to participate in society expressed in our study. Furthermore, awareness of statelessness on the part of Canadian officials (and members of society) can alleviate the burden of explaining one’s legal predicam-ent to multiple immigration authorities, which contributed to levels of frustration, anxiety, and hopelessness. Hence, recognition could facilitate the sense of equal membership with peers, family members and society, promote dignity and personhood, and limit experiences of liminality.

Second, considering that solutions “cannot be reduced to a legalistic or medicalized analysis — but [require] an appreciation of social theory and, ideally, social justice” (Sköld 2023, 2), we call for an institutional response that considers statelessness holistically and acknowledges diversity in causes — from in situ to migratory contexts — and responds with context-tailored remedies. Such a response should be institutionalized, for instance, within the Immigration and Refugee Board (IRB) and Immigration, Refugees, Citizenship Canada (IRCC) rather than applied through a case-by-case ministerial granting process. In this way, statelessness would move from an exception at the margins of Canadian immigration and nationality legislation and policy to join the already well-established protection infrastructure for other non-citizen groups.

Delineating the technical parameters of such a response is outside the scope of this paper for two key reasons. First, the IRB and IRCC should inform the public on whether already internally established statelessness determination procedures exist in Canada, and whether these procedures are effective in identifying and protecting stateless persons outside of the context of asylum. Second, our study illustrates that the dividing line between in situ and migratory statelessness is difficult to draw, and legal status is not static. Additionally, all of our participants faced significant hurdles in obtaining birth registration or proving the validity of or renewing their identity documents. Thus, comprehensive research is needed on the specific causes of statelessness among Canadian residents, including Indigenous populations (see Kane 2019). A detailed understanding of causes and of existing responses can better inform what a holistic SDP could look like in the Canadian context.

Learning about the health care experiences from those directly affected, we hope to have provided valuable evidence to a diverse group of discussants of legislative and policy reform, including activists, academics, policy makers, legislators, service provider organizations, and stateless people themselves.

## Footnotes

1 The right to health does not equal the right to be healthy, as a state cannot ensure good health and cannot protect against every possible cause of ill health. See CESCR 2000, para 8 and 9.

2 The term non-citizens is broad and includes, inter alia, asylum-seekers, refugees, permanent residents, and stateless persons, groups which may overlap and intersect, despite specific experiences and needs in society. We employ the term legal status to refer to this plethora of statuses which includes statelessness.

3 See UNHCR 2020; Bianchini 2017.

4 Though Immigration, Refugees, and Citizenship Canada (IRCC) recently defined *de jure* and *de facto* statelessness in internal policy guidance for determining applications for permanent residency on humanitarian and compassionate grounds (see Government of Canada 2017).

5 Figures range. See Kane 2019.

6 A work permit can be granted to a foreign national who is awaiting refugee status determination or who is subject to an unenforceable removal order (s. 206(1), Government of Canada n.d.-c).

7 See Government of Canada 2023a.

8 For example, the CESCR called on Canada to provide information on measures taken to identify, reduce, and prevent statelessness, as well as data on determinants of health including “the availability and accessibility of employment, social security, housing, health-care services and education” (CESCR 2020). See also UNGA (2018).
